# Siglec Receptors Modulate Dendritic Cell Activation and Antigen Presentation to T Cells in Cancer

**DOI:** 10.3389/fcell.2022.828916

**Published:** 2022-03-03

**Authors:** Jinyu Wang, Michela Manni, Anne Bärenwaldt, Ronja Wieboldt, Nicole Kirchhammer, Robert Ivanek, Michal Stanczak, Alfred Zippelius, David König, Natalia Rodrigues Manutano, Heinz Läubli

**Affiliations:** ^1^ Department of Biomedicine, University Hospital and University of Basel, Basel, Switzerland; ^2^ Swiss Institute of Bioinformatics, Basel, Switzerland; ^3^ Division of Oncology, Department of Theragnostic, University Hospital Basel, Basel, Switzerland

**Keywords:** sialic acid, tumor immunology, glyco-immune checkpoint, antigen, antigen processing

## Abstract

Interactions between sialylated glycans and sialic acid-binding immunoglobulin-like lectin (Siglec) receptors have been recently described as potential new immune checkpoint that can be targeted to improve anticancer immunity. Myeloid cells have been reported to express a wide range of different Siglecs; however, their expression and functions on cancer-associated dendritic cells (DCs) were not fully characterized. We found that classical conventional DCs (cDCs) from cancer patient samples have a high expression of several inhibitory Siglecs including Siglec-7, Siglec-9, and Siglec-10. In subcutaneous murine tumor models, we also found an upregulation of the inhibitory Siglec-E receptor on cancer-associated cDCs. DC lines and bone marrow-derived DCs (BMDCs) with expression of these inhibitory Siglecs showed impaired maturation states on transcriptome and protein level. Furthermore, ablation of these inhibitory Siglecs from DCs enhanced their capability to prime antigen-specific T cells and induce proliferation. Our work provides a deeper understanding of the influence of inhibitory Siglecs on DCs and reveals a potential new target to improve cancer immunotherapy.

## Introduction

The treatment of cancer with immune checkpoint inhibitors (ICI) has significantly improved the prognosis of cancer patients (Topalian et al., 2015; Zou et al., 2016; Chen and Mellman, 2017; Ribas and Wolchok, 2018). However, most patients respond only shortly or not at all to ICI treatment. Therefore, a better understanding of immunosuppression status during cancer progression is of importance for enhanced therapeutic efficacy. Tumor cell hypersialylation has been reported as one of the tumor-intrinsic factors dampening anti-tumor immunity (Fraschilla and Pillai, 2017). Aberrant glycosylation status in the tumor micro-environment (TME) is associated with tumor invasion and metastasis (Hakomori, 1996). Sialoglycans derived from altered glycosylation can be recognized by inhibitory sialic acid-binding immunoglobulin-like lectins (Siglecs), characterized by their intracellular immunoreceptor tyrosine-based inhibitory motifs (ITIM) (Crocker et al., 2007; Macauley et al., 2014; Nitschke, 2014; Varki et al., 2015; Pearce and Läubli, 2016). Cancer cells are reported to exploit this mechanism as a survival strategy, through overexpression of sialyltransferases, scavenging of food-derived Neu5Gc, or expression of 9-O-acetyl-GD3 (Tangvoranuntakul et al., 2003; Kniep et al., 2006; Hedlund et al., 2008; Pearce and Läubli, 2016).

Recent studies demonstrated that several inhibitory Siglecs are involved in immunosuppression of T cells, NK cells, neutrophils, and macrophages (Läubli et al., 2014a; Läubli et al., 2014b; Hudak et al., 2014; Jandus et al., 2014; Beatson et al., 2016; Stanczak et al., 2018; Barkal et al., 2019; Wang et al., 2019). Dendritic cells (DCs) play a central role as antigen-presenting cells in the tumor tissue and draining lymph nodes to prime tumor-specific T cell response (Broz et al., 2014; Roberts et al., 2016; Salmon et al., 2016; Spranger et al., 2017; Binnewies et al., 2019). Previous reports have demonstrated that Siglec receptors can impact DC function (Ding et al., 2016; Perdicchio et al., 2016; Silva et al., 2016). For example, inhibitory Siglec-G on DCs has been linked to inhibition of antigen cross-presentation in murine models (Ding et al., 2016). Moreover, interactions of sialic acid-containing carbohydrates on antigens with Siglec-E can modulate DC activation (Perdicchio et al., 2016). However, the expression and function of inhibitory Siglecs on cancer-associated DCs is still not well understood. Here, we study the expression of human and murine Siglec receptors in cancer and the functional implication of such receptors on DCs.

## Materials and Methods

### Patient Samples

The local ethics committee in Basel, Switzerland, approved the sample collection and the use of the corresponding clinical data (Ethikkommission Nordwestschweiz, EKNZ, Basel Stadt, Switzerland, EKNZ 2018-01990). Informed consent was obtained from all patients prior to sample collection. Tumor samples were collected locally at the University Hospital Basel, digested, and processed, and single-cell suspensions were frozen. Human peripheral blood mononuclear cells (PBMCs) were isolated by density gradient centrifugation from buffy coats obtained from University Hospital Basel. Single-cell suspension of PBMCs were frozen in liquid nitrogen.

### Animal Strains

Siglec-E^flox^ mice were generated in collaboration with Biocytogen Company and crossed with CD11c-cre mice kindly provided by Prof. Daniela Finke. Siglec-E systemic knockout (EKO) mice was obtained from the group of Prof. Ajit Varki. Siglec-9 transgenic mice were previously reported (Läubli et al., 2014b). To generate a high frequency of Siglec-9-expressing mouse bone marrow-derived DCs (BMDCs), Siglec-9^flox^ mice were crossed with XCR1-cre mice. All mouse experiments were approved by the local ethics committee (Basel Stadt, Switzerland) and performed in accordance with the Swiss federal regulations.

### Cell Lines

Mouse colorectal cancer cell line MC38 was kindly provided by our collaborator from Hannover. tdTomato-expressing MC38 cell line was generated by our lab through lentiviral transduction, with the Luc2-tdTomato plasmid kindly provided by Prof. Gregor Hutter. OVA-expressing MC38 (MC38-OVA) cell line was kindly provided by Prof. Mark Smyth. Chinese Hamster Ovary cell line with FMS-like tyrosine kinase 3 ligand secretion capability (CHO-Flt3L) was kindly provided by Dr. Panagiotis Tsapogas. Mouse immature dendritic cell line Sp37A3 was kindly provided by Merck KGaA.

### Cell Line Culture

Mouse cancer cell lines were maintained in Dulbecco’s Modified Eagle Medium (Sigma, United States) supplemented with 10% heat-inactivated fetal bovine serum (PAA Laboratories, Germany), 1 mM sodium pyruvate (Gibco, United States), 1× MEM non-essential amino acid solution (Sigma, United States), and 100 μg/ml streptomycin and 100 U/ml penicillin (Gibco, United States).

CHO-Flt3L cells were maintained in Iscove’s Modified Dulbecco’s Medium (Sigma, United States) supplemented with 5% heat-inactivated fetal bovine serum (PAA Laboratories, Germany).

Sp37A3 mouse dendritic cell line and relative genetically modified lines were maintained in Iscove’s Modified Dulbecco’s Medium (Sigma, United States) supplemented with 10% heat-inactivated fetal bovine serum (PAA Laboratories, Germany), 1 mM sodium pyruvate (Gibco, United States), 1× MEM non-essential amino acid solution (Sigma, United States), 100 μg/ml streptomycin and 100 U/ml penicillin (Gibco, United States), 0.05 mM 2-mercaptoethanol (Gibco, United States), 20 ng/ml recombinant mouse GM-CSF (Peprotech, United Kingdom), and 20 ng/ml recombinant mouse M-CSF (Peprotech, United Kingdom).

### Mice Primary Bone Marrow Cell Culture

Mouse BMDCs were generated by plating five million bone marrow cells freshly isolated from the tibia and femur into 10-cm dishes. During the 7-day cultivation, the bone marrow cells were maintained in RPMI-1640 Medium (Sigma, United States) supplemented with 10% heat-inactivated fetal bovine serum (PAA Laboratories, Germany), 1 mM sodium pyruvate (Gibco, United States), 1× MEM non-essential amino acid solution (Sigma, United States), 100 μg/ml streptomycin and 100 U/ml penicillin (Gibco, United States), 0.05 mM 2-mercaptoethanol (Gibco, United States), and 10 ng/ml mouse GM-CSF (Peprotech, United Kingdom) in several first experiments.

### Animal Tumor Models

For tumor-bearing mice experiments, 7–12 weeks old mice were used. Then, 5 × 10^5^ tumor cells were injected subcutaneously into the right thoracic flank. Tumor size and health score were measured and monitored three times per week. Perpendicular tumor diameters were measured by a caliper and tumor volume calculated according to the following formula: tumor volume = (*d* ^2^ × *D*)/2, where *d* and *D* represent the shortest and longest diameters of the tumors (in millimeter), respectively. For tumor growth experiments, mice were sacrificed once tumor size reached 1,500 mm^3^. For tumor-infiltrating DC phenotype and functionality experiments, mice were sacrificed once tumor size reached 300–500 mm^3^.

### Tumor Digests and PBMC Isolation

For the preparation of single-cell suspensions from both human and mouse tumors, tumors were collected, and surgical specimens were mechanically dissociated and subsequently digested using accutase (PAA Laboratories, Germany), collagenase IV (Worthington, United States), hyaluronidase (Sigma, United States), and DNase type IV (Sigma, United States) for 1 h at 37°C under constant agitation. Cell suspensions were filtered through 70-µm mesh twice and lysed for red blood cells using RBC lysis buffer (eBioscience, United States). PBMCs were isolated by density gradient centrifugation using Histopaque-1077 (Sigma, United States) from buffy coats. Mice splenocytes were isolated by mechanical disruption using the end of a 1-ml syringe, lysed for red blood cells using RBC lysis buffer, then digested with Collagenase D (Roche, Switzerland) and DNase I (Roche, Switzerland). Samples were either used directly or frozen (in 90% FBS, 10% DMSO) and stored in liquid nitrogen until the time of analysis.

### Generation of Siglec-E Knockout Sp37A3 Cells

Siglec-E-deficient Sp37A3 cells were generated by using CRISPR/Cas9-mediated gene editing. Guide RNAs were designed online based on published data (http://greenlisted.cmm.ki.se/). Guide RNAs with the following sequences were synthesized by Microsynth (Switzerland): forward: 5′—CAC CGG AGG GTC AGA ACC CCC AAG—3′; reverse: 5′—AAA CCT TGG GGG TTC TGA CCC TCC—3′. Then, they were cloned into the lentiCRISPRv2 puro vector (Addgene plasmid #98290). Lentivirus with empty vectors or modified vectors were used to transduce the original Sp37A3 cell line. Single-cell clones with right phenotype were sorted into 96-well plates. After their recovery and expansion, individual clones were screened again for Siglec-E expression. Multiple clones were selected and pooled to avoid clonal selection.

### Genetically Modified Sp37A3 Cell RNA Sequencing Analysis

Control empty vector-transduced (CtrV) and EKO Sp37A3 cells were taken from culture. Then, 1 × 10^6^ cells were seeded in six-well plates and pulsed with 0.1 mg/ml EndoFit Ovalbumin (Invivogen, United States) for 2 h. Then, cells were washed and stimulated for maturation by 0.1 ug/ml lipopolysaccharides (Sigma, United States) for 24 h. Cells were washed, and RNA samples were prepared by RNeasy Plus Micro Kit (Qiagen, Germany). The cDNA library was prepared, and next-generation sequencing of the library and data analysis was performed *n*th. Reads were aligned to the mouse genome (UCSC version mm10) with STAR (version 2.7.0c) with default parameters except for allowing up to 10 genome hits (outFilterMultimapNmax 10), reporting only one location for hits with equal score (outSAMmultNmax 1), and for filtering reads without evidence in spliced junction table (outFilterType “BySJout”). The output was sorted and indexed with SAMtools (v 1.9). Read and alignment quality was evaluated using the qQCReport function of the Bioconductor package QuasR (v 1.30.0). The featureCounts function from Bioconductor package Rsubread (v 2.4.3) was used to count the number of reads (5ʹ ends) overlapping with the exons of each gene assuming an exon union model (with used gene model provided by Ensembl v101). The data were normalized by applying the TMM method from Bioconductor edgeR package (version 3.32.1). Only genes having log2 CPM counts bigger than 0 in at least two samples were kept for further analyses. The principal component analysis was based on 25% of most variable genes in the dataset. The differentially expressed genes were identified using the quasi-likelihood (QL) method implemented in edgeR package (version 3.32.1) using replicate ID as covariate. Genes with FDR smaller than … and minimum log2 fold change of … were used and considered as differentially expressed.

### Neuraminidase Treatment and Maturation Analysis

CtrV and EKO Sp37A3 cells were seeded and stimulated for 24 h with sialidase. Prior to the stimulation, CtrV and EKO Sp37A3 cells were treated with 10 mU/ml neuraminidase from *Vibrio cholerae* (VCN, Sigma) for 30 min. Maturation was measured by analysis of MHC II expression.

### CtrV and EKO Sp37A3 Cell Cytokine/Chemokine Array Analysis

CtrV and EKO Sp37A3 cells were seeded and pulsed. After 36 h of LPS stimulation, culture supernatant was collected, frozen, and sent in dry ice for a 44-plex Cytokine/Chemokine Array test (Eve Technologies, Canada). Cytokine and chemokine concentrations were analyzed and presented by Eve Technologies (Canada).

### DC and Antigen-Specific T Cell Co-Culture

BMDCs or Sp37A3 cells were seeded 4 × 10^4^ cells per well in 96-well plate. Then, cells were pulsed with 0.1 mg/ml OVA protein (Invivogen, United States) or left unpulsed for 2 h. DCs were washed and stimulated by 0.1 µg/ml LPS for overnight. OVA antigen-specific OT-I CD8^+^ T cells and OT-II CD4^+^ T cells were isolated from spleens of indicated mice, respectively, by MACS (Stemcell, Canada). T cells were labelled with CellTrace Violet (CTV, Invitrogen, United States) and added into wells, at 2 × 10^5^ cells per well. T cell activation and proliferation was checked after certain timepoints as described in each experiment.

### Multicolor Flow Cytometry

For multicolor flow cytometry, dead cells and doublets were excluded in all analyses (also Supplementary Figure S1). Corresponding isotype antibodies or fluorescence-minus-one (FMO) samples were used as a control, in particular for the Siglec stainings. All tumor samples were analyzed with a Fortessa LSR II flow cytometer (BD Biosciences). For infiltration analysis, mice were euthanized, and tumors were mechanically dissociated and digested as described for the human sample preparation.

### Statistical Analysis

Statistical analysis was performed using Prism 9 (GraphPad, United States). Different comparison strategies were indicated in each specific figure respectively.

## Results

Tumor-infiltrating conventional dendritic cells express inhibitory Siglec receptors in humans.

**FIGURE 1:**
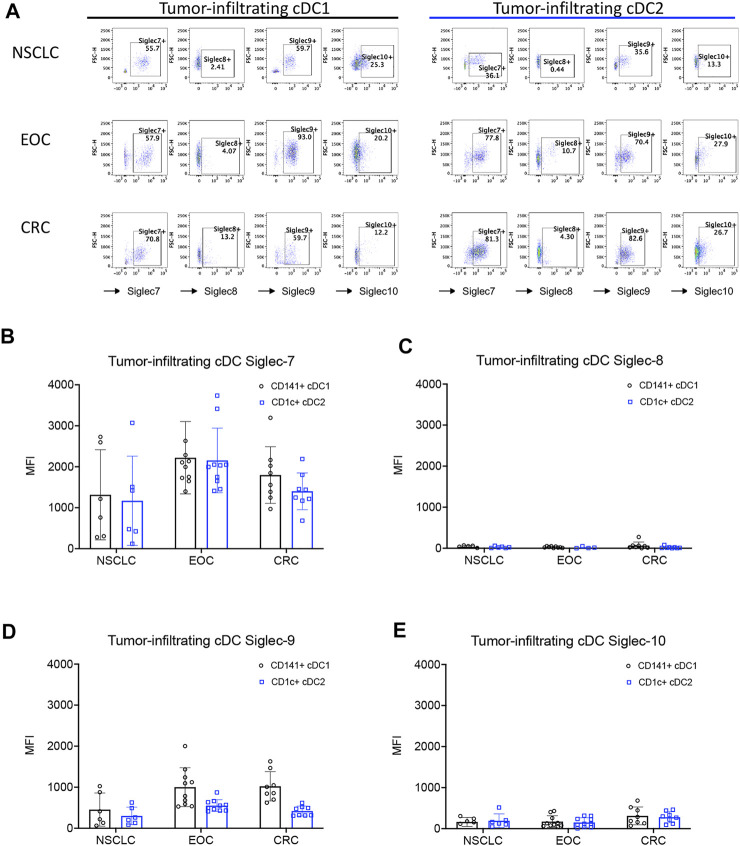
Expression of inhibitory Siglecs on human tumor-infiltrating DCs. **(A)** Representative examples of Siglec receptor expression determined by flow cytometry on different cDC subsets from primary patient samples. **(B–E)** The expression levels of inhibitory Siglecs on DCs from primary tumor samples of patients with non-small cell lung cancer (NSCLC), epithelial ovarian cancer (EOC), and colorectal cancer (CRC). Data are presented as mean ± SD.

Previous reports have shown that Siglec receptors are expressed on myeloid cells, including cDCs (Chen et al., 2009; Ding et al., 2016; Fraschilla and Pillai, 2017; Barenwaldt and Laubli, 2019; Ruffin et al., 2019). However, the expression and functions of these receptors on intratumoral cDCs from patients with different types of cancer are poorly understood. Therefore, we tested the expression of several inhibitory Siglec receptors on tumor-infiltrating conventional DCs (Ti-cDCs) from different types of cancers including epithelial ovarian cancer (EOC), non-small cell lung cancer (NSCLC), and colorectal cancer (CRC) by flow cytometry (Figure 1A and Supplementary Figures S1A, S2). We found a significant proportion of both type 1 and type 2 cDC-expressing inhibitory Siglec receptors. Across the different cancer types tested, Siglec-7 and Siglec-9 were consistently expressed by a higher percentage of Ti-cDCs compared to other Siglecs. Siglec-10 showed low to intermediate expression levels on Ti-cDCs, while Siglec-8 expression was even less frequent. The results of geometric mean fluorescence intensity (MFI) for each inhibitory Siglecs also revealed higher expression levels of Siglec-7 and Siglec-9 compared to the other two Siglecs (Figures 1B–E; Supplementary Figures S1, S2). Similar expression patterns of these Siglec receptors were also observed on plasmacytoid dendritic cells (pDCs), although to a lower percentage (Supplementary Figure S3). Taken together, this data demonstrates that inhibitory Siglecs are expressed on human cancer-associated DCs and could be potentially involved in the regulation of these cells.

### Siglec-E Expression Is Upregulated on Intratumoral DCs

**FIGURE 2:**
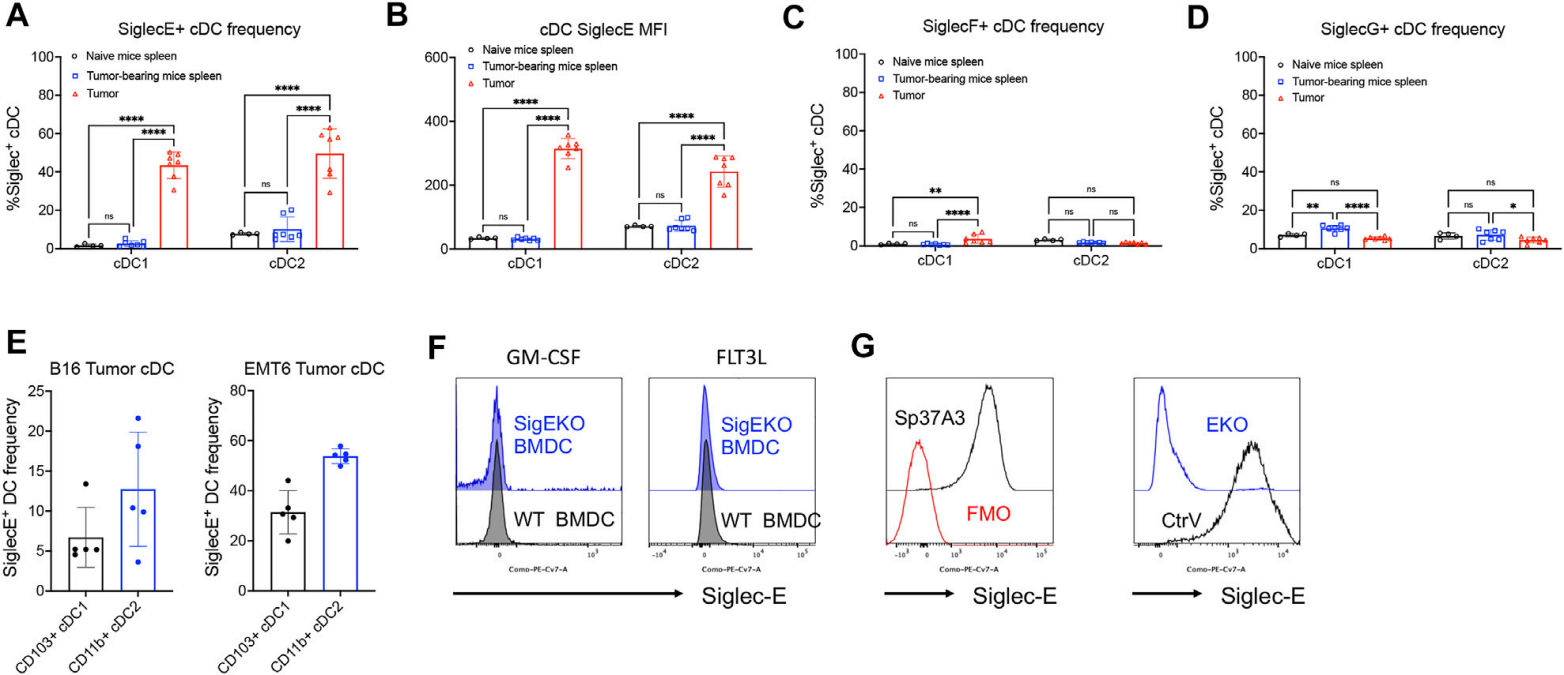
Inhibitory Siglec-E expression is significant on mouse tumor-associated DCs. **(A–D)** The expression patterns of several murine inhibitory Siglecs on cDCs isolated from naive C57BL/6 mice spleens, MC38 tumor-bearing mouse spleens, and primary subcutaneous tumors were analyzed by flow cytometry. **(A)** SiglecE + cDC frequency, **(B)** SiglecE MFI, **(C)** SiglecF + cDC frequency, and **(D)** SiglecG + cDC frequency. **(E)** Siglec-E expression on tumor cDC subsets from B16 melanoma and EMT6 breast cancer mouse models. **(F)** Siglec-E expression of BMDCs from wildtype (WT, black line) and systemic Siglec-E knockout (EKO, blue line) mice after 7-day *in vitro* culture supplemented with GM-CSF or FLT3L. **(G)** The expression of Siglec-E on Sp37A3 cell line (black line) versus FMO control (red line). Siglec-E expression of Siglec-E knockout (EKO) Sp37A3 cells and empty control vector (CtrV)-transduced Sp37A3 cells. Data are presented as mean ± SD, and two-way ANOVA was used for two-way comparisons (*p < 0.0332, **p < 0.0021, ***p < 0.0002, and ****p < 0.0001).

To further investigate the function of Siglec receptors on cDCs during cancer progression, we next analyzed the expression of several inhibitory Siglec receptors in mice. DCs were isolated from the spleen of healthy C57BL/6 wildtype mice or from the spleen and tumor tissue of MC38 tumor-bearing mice. The expression of inhibitory Siglec-E, -F, and -G was analyzed by flow cytometry (Figures 2A–D; Supplementary Figure S4). We observed that these inhibitory Siglecs were only expressed by very small proportions of spleen cDCs from either naïve mice or MC38 subcutaneous tumor-bearing mice. However, Siglec-E expression, but not Siglec-F and Siglec-G, was quite pronounced on both Ti-cDC subsets, suggesting a unique role of this molecule on DC biology or functions during tumor progression (Figures 2A,B). The expression of Siglec-E on Ti-cDCs was also confirmed in two other murine tumor models, including the B16 melanoma and the EMT6 breast cancer model in C57BL/6 and BALB/c mice, respectively (Figures 2E,F).

### Siglec-E-Deficient DCs Are More Responsive to Stimulation

**FIGURE 3:**
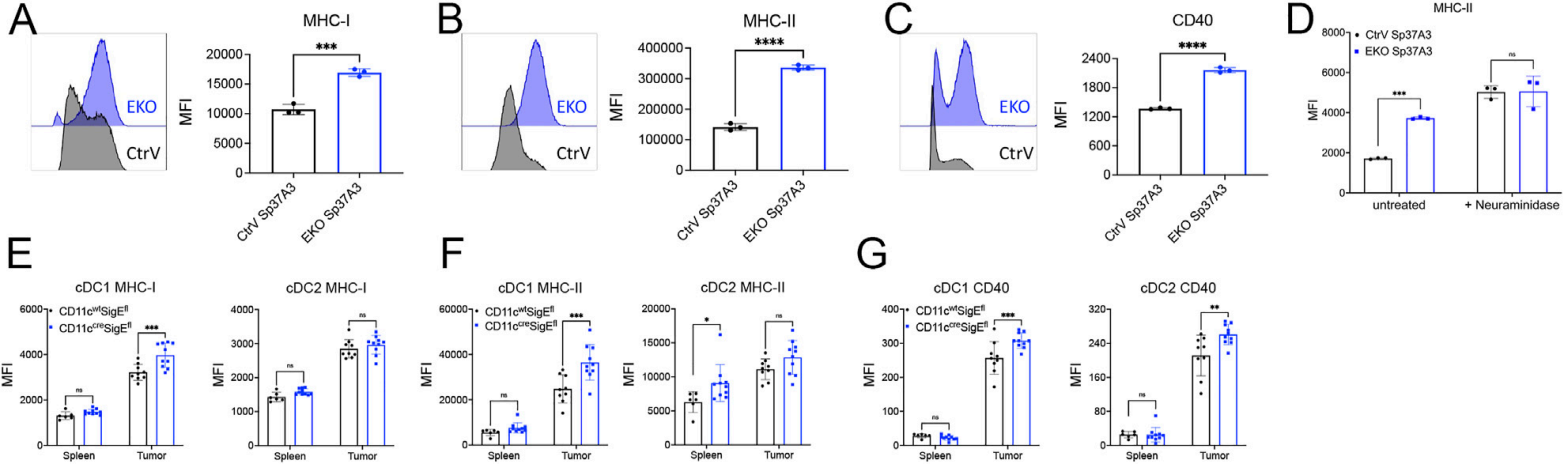
Siglec-E-deficient DCs showed enhanced phenotypic maturation. **(A–C)** Flow cytometry analysis of the expression levels of several DC maturation markers on CtrV Sp37A3 cells (black) and EKO Sp37A3 cells (blue), including MHC-I **(A)**, MHC-II **(B)**, and CD40 **(C)**. **(D)** treatment of CtrV Sp37A3 cells (black) and EKO Sp37A3 cells (blue) with neuraminidase and maturation with LPS was measured by the expression of MHC II. **(E–G)** Maturation markers on spleen and tumor-infiltrating cDCs isolated from MC38 subcutaneous tumor models of CD11c^cre^SigE^fl^ mice (blue) and littermates (CD11c^wt^SigE^fl^, black) by flow cytometry. Data are presented as mean (±SD). Two-way ANOVA was used for two-way comparisons, and unpaired *t* test was used for one-way comparisons (*p < 0.0332, **p < 0.0021, ***p < 0.0002, and ****p < 0.0001).

To explore the possible function of Siglec-E expression on DCs in an *in vitro* system, we next analyzed the expression of Siglec-E on BMDC. Neither GM-CSF- nor FLT3L-derived BMDC expressed significant Siglec-E levels as compared to cells derived from Siglec-E-deficient mice (Figure 2G). We then screened several murine DC cell lines for Siglec-E expression. Among them, an immature DC cell line Sp37A3, generated from C57BL/6 mouse spleen (Bros et al., 2007), showed significant expression of Siglec-E (Figure 2H). Thus, we next used a CRISPR-Cas9-based lentivirus transduction system to deplete *Siglece* in Sp37A3 cells (EKO Sp37A3 cells). After pooling several single clones that were confirmed to have minimal or no Siglec-E expression, we successfully generated EKO Sp37A3 line, along with an empty CtrV Sp37A3 line (Figure 2H). We then directly explored whether Siglec-E influences DC activation and maturation. We first analyzed markers of DC maturation on the newly generated CtrV Sp37A3 and EKO Sp37A3 cell lines. The DC phenotypic maturation markers we investigated, including MHC-I, MHC-II, and CD40, all showed significant upregulation on EKO Sp37A3 cells compared to the Siglec-E-expressing Sp37A3 cells (Figures 3A–C). We further investigated how treatment with neuraminidase influences activation of CtrV Sp37A3 and EKO Sp37A3 cell lines. Indeed, desialylation led to an increased maturation and MHC II expression on the DC cell lines (Figure 3D). To confirm these findings *in vivo*, we generated CD11c^cre^SigE^flox/flox^ mice (ELOX mice) that display specific SiglecE deficiency from the CD11c-expressing cells with the cre recombinase, affecting mainly cDCs. As expected, naïve spleen cDCs from ELOX mice and their littermates do not show significant differences in maturation (Supplementary Figure S5). However, similar to the *in vitro* data, intratumoral cDCs showed a significant increase of MHC-I, MHC-II, and CD40 markers on tumor-infiltrating cDC1 cells lacking Siglec-E compared to cDC1 cells from littermate control mice (Figures 3D–F). Only CD40 was significantly increased in cDC2 from ELOX mice but not MHC-I and MHC-II (Figures 3D–F). Taken together, our newly generated Sp37A3 cell lines appeared to be appropriate models to study the roles of Siglec-E on cDCs.

### Siglec-E Regulates DC Activation and Cytokine Secretion

**FIGURE 4:**
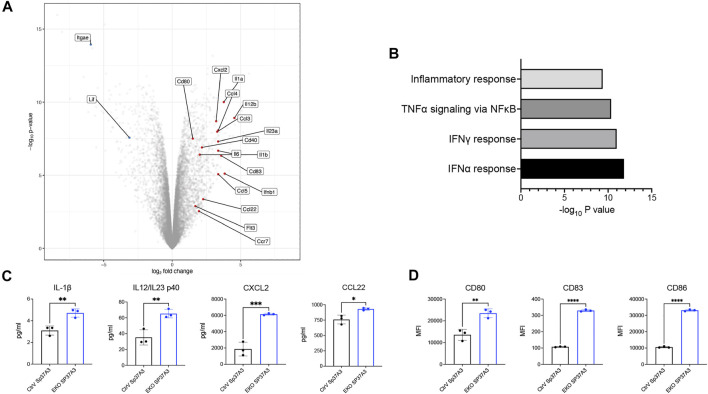
Siglec-E-deficient DCs showed elevated maturation status upon stimulation. **(A)** Volcano plot of differentially expressed genes between EKO SP37A3 and CtrV Sp37A3 cells. **(B)** GSEA of the most significantly activated pathways in EKO Sp37A3 cells. **(C)** Cytokine and chemokine production and **(D)** activator and co-stimulatory markers of CtrV (black) and EKO (blue) Sp37A3 cells. Data are presented as mean (±SD), and unpaired *t* test was used for one-way comparisons (*p < 0.0332, **p < 0.0021, ***p < 0.0002, and ****p < 0.0001).

As Sp37A3 cell line was reported as an immature cell line (Bros et al., 2007), we next investigated how our newly generated cell lines respond to antigen and maturation stimuli. We compared the transcriptional profile of OVA-pulsed, LPS-stimulated CtrV and EKO Sp37A3 by bulk RNA sequencing. The EKO Sp37A3 cells upregulated the mRNA levels of co-stimulatory molecules, multiple chemokines, and cytokines including CD80, CD40, CCL2, CCL4, CCL5, and IL-23 (Figure 4A). Gene Set Enrichment Analysis (GSEA) suggests that several pathways were differentially activated in cells lacking Siglec-E (Figure 4B). In particular, EKO Sp37A3 cells showed stronger upregulation of type I and II interferon (IFN-α and IFN-γ)-related responses, tumor necrosis factor alpha (TNF-α) response, and general inflammatory response (Figure 4B). To validate these findings at protein level, we collected cell culture supernatants of OVA-pulsed, LPS-stimulated Sp37A3 cells and performed a Mouse Cytokine/Chemokine Array assay. Among the 44 cytokines and chemokines, the levels of interleukin (IL)-1β, IL-12, or IL-23 p40 subunit (IL12/IL23 p40); CXCL2; and CCL22 showed different secretion patterns between the two Sp37A3 lines (Figure 4C and Supplementary Figure, S6A,B). Similar to the *in vitro* and *in vivo* data, analysis of the surface maturation markers and co-stimulatory molecules on protein level also showed that EKO Sp37A3 cells showed and increased activation profile compared to CtrV Sp37A3 cells (Figure 4D and Supplementary Figure S6C). Taken together, these results indicate that the EKO Sp37A3 show a higher activation and maturation status upon stimulation compared to the Siglec-E expressing control cell line. This data suggests that Siglec-E can modulate cDC activation and potentially also influence antigen presentation.

### Inhibitory Siglecs Modulate Antigen Presentation

**FIGURE 5:**
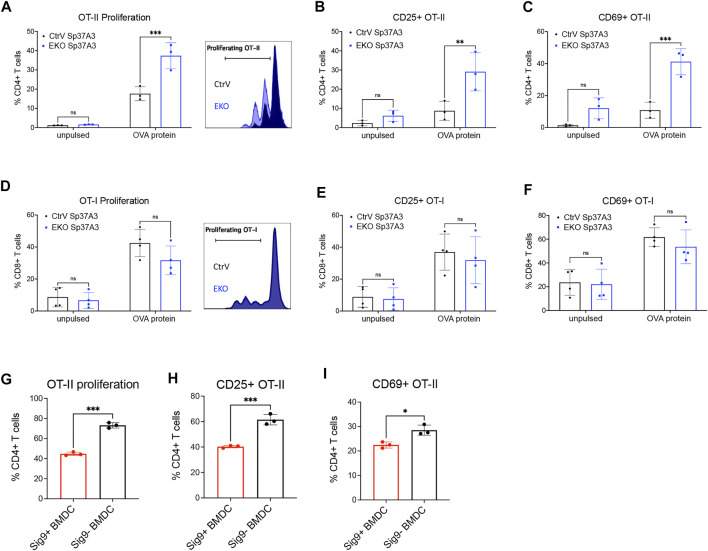
Inhibitory Siglecs impair DC antigen presentation to CD4^+^ T cells. **(A–F)** 48-h *in vitro* co-culture of OVA-pulsed CtrV or EKO Sp37A3 cells with **(A–C)** CD4^+^ OT-II T cells or **(D–F)** CD8^+^ OT-I T cells. Data was pooled from several independent experiments, and each datapoint represents the average of the technical replicates in individual experiments. **(G–I)** 72-h *in vitro* co-culture of OVA-pulsed Siglec-9-positive (red) or Siglec-9-negative (black) BMDCs with CD4^+^ OT-II T cells. Data are presented as mean (±SD). Two-way ANOVA was used for two-way comparisons, and unpaired *t* test was used for one-way comparisons (*p < 0.0332, **p < 0.0021, ***p < 0.0002, and ****p < 0.0001).

In order to understand whether the antigen presentation of cDCs is affected by the expression of Siglec-E, we studied antigen handling including uptake, processing, and presentation in Sp37A3 cells. First, we analyzed antigen endocytosis of the Sp37A3 cells with fluorescent-labelled soluble OVA antigen or tumor cell-associated antigens. No difference of antigen uptake was observed between EKO and control cDCs (Supplementary Figures S7A,B). Furthermore, we co-cultured the Sp37A3 cells with live fluorescent-labelled or GFP-expressing MC38 tumor cells. Neither the frequency of fluorescent-positive DCs nor the MFI showed any significant change (Supplementary Figures S7C,D). As mannose receptor (MR) was reported to be the key mediator of soluble OVA antigen uptake by DCs (Burgdorf et al., 2006), we also examined its expression levels on both Sp37A3 cells. However, we observed even less MR expression on the EKO Sp37A3 cells, suggesting MR is of less importance in our scenario (Supplementary Figure S7E). Since antigen uptake was not affected by the Siglec-E expression, we then investigated whether Siglec-E expression affects antigen processing. We pulsed EKO Sp37A3 and control Sp37A3 cells with DQ-OVA and followed processing by measuring the fluorescent signal. We measured the fluorescence at several timepoints and found that the EKO Sp37A3 DCs showed a stronger fluorescent signal compared to control Sp37A3 cells, suggesting a more efficient antigen processing (Supplementary Figure S7F). To understand whether this leads to better antigen presentation, we used OVA-pulsed Sp37A3 cells to co-culture with either antigen-specific T cells from OT-I and OT-II transgenic mice or T cell proliferation was analyzed after CTV staining, and activation was studied by CD25 and CD69 expression on T cells. Even though both MHC-I and MHC-II molecules are expressed at higher levels in EKO Sp37A3 cells, mainly OT-II CD4^+^ T cells showed better activation and proliferation during co-culture (Figures 5A–F). As sialic acids were previously reported to favor regulatory T cell (Tregs) polarization of naïve OT-II cells (Perdicchio et al., 2016), we tested the frequency of Foxp3+ Tregs at the end of the co-culture time and found only very low frequency of the CD4^+^ OT-II cells differentiated into Tregs (Supplementary Figure S7G). We also tested the potential effect on cross-presentation by using heat-shocked wildtype or OVA-expressing MC38 tumor cells (MC38-wt or MC38-OVA) to replace soluble OVA antigen. Heat-shocked MC38-OVA cells induced a strong OT-I CD8^+^ T cell activation and proliferation, but no difference was observed between the two Sp37A3 lines (Supplementary Figures S7H–J). Furthermore, differences between cell lines were still visible when not maturated with LPS (Supplementary Figure S8). To investigate whether the overexpression of an inhibitory Siglec would influence DC antigen presentation, we used the Siglec-9 transgenic mouse crossed to CD11c-Cre mice (Läubli et al., 2014b; Stanczak et al., 2018). Unlike mouse Siglec-E, naive BMDCs generated from these transgenic mice showed expression of human Siglec-9. Similar to our observations on Siglec-E-expressing DCs, OT-II CD4^+^ T cells co-cultured with OVA antigen-pulsed Siglec-9-positive BMDCs represented less activation and proliferation (Figures 5G–I). Taken together, these results suggested that the expression of inhibitory Siglecs could modulate antigen processing and presentation to CD4^+^ T cells.

## Discussion

In this work, we show that inhibitory Siglec receptors are expressed on DCs in cancer patients and can modulate DC activation and antigen presentation to T cells. Although several inhibitory Siglecs have been reported to influence myeloid cell functions in cancer, the understanding of the role of Siglec receptors on DCs in particular in the context of cancer is rather limited (Bax et al., 2007; Ding et al., 2016; Perdicchio et al., 2016; Silva et al., 2016). In our analysis, we found that several inhibitory Siglecs are observed on cDCs from human cancer patients and in murine tumor models. In various patient biopsies from different cancer types, we detected a high expression of several inhibitory Siglecs on Ti-cDCs, in particular Siglec-7 and Siglec-9. Also, in different tumor models, we found an increased expression of Siglec-E, which is a functional paralog of human Siglec-9. Previous works have described Siglecs on DCs (Bax et al., 2007; Chen et al., 2009; Kawasaki et al., 2014; Ding et al., 2016; Affandi et al., 2020; Perez-Zsolt et al., 2021). For example, Siglec-7 has been targeted on human monocyte-derived DCs to deliver antigen (Kawasaki et al., 2014). In another study, Siglec-10 (and Siglec-G in mice) had been demonstrated to influence the response to damage-induced stress (Chen et al., 2009).

In order to study the functional role of inhibitory Siglec receptors on DCs, we tried to use BMDCs from wildtype and Siglec-E-deficient mice. However, although myeloid bone marrow cells express a high level of Siglec-E, BMDCs are not expressing Siglec-E. Previous studies have also found similar results when comparing to complete Siglec-E-deficient animals (Nagala et al., 2017). We therefore screened several mouse DC cell lines for functional testing. The C57BL/6 background mouse spleen-derived DC cell line Sp37A3 expresses high level of Siglec-E. By comparison of Siglec-E-deficient (EKO) and control (CtrV) Sp37A3 DCs, we found that Siglec-E ablation increased DC maturation. This *in vitro* finding was confirmed on mouse tumor-infiltrating DCs utilizing a conditional Siglec-E knockout mouse model. Further examination by transcriptomic analysis showed an elevated secretion of cytokines and chemokines, as well as surface co-stimulatory molecules. In a previous work, the loss of Siglec-G led to an increased response to damage-associated signaling molecules including high mobility group box 1 (HMGB1), IL-6, and TNF-⍺ (Chen et al., 2009). The lack of Siglec-G on DCs also led to improved cross-presentation of antigens (Ding et al., 2016). In the same work, Siglec-G-deficient DCs also led to an improved anti-tumoral immune response (Ding et al., 2016). In our study, the lack of Siglec-E had no direct influence on cross-presentation but led to an increased DC activation, enhanced antigen processing, and induced a stronger T cell proliferation, predominantly in CD4^+^ T cells. A previous work has demonstrated that increased lysosomal processing could influence MHC II presentation while not so much modulating MHC I presentation (Samie and Cresswell, 2015). We found an increased processing when Siglec-E was deleted in DC cell lines, which could potentially explain the difference on MHC I and MHC II presentation and T cell stimulation. The role of inhibitory Siglecs on MHC II-mediated antigen presentation was also confirmed by a mouse BMDC model, which was engineered to express human Siglec-9. The Siglec-9-expressing BMDCs showed a worse antigen-specific CD4^+^ T cell activation, demonstrating that the overexpression of an inhibitory Siglec receptor can inhibit antigen presentation and T cell activation.

Interactions between Siglec receptors and sialoglycans have been recently described as a potential new immune checkpoint to improve anti-cancer immunity, in particular on innate immune cells (Beatson et al., 2016; RodrÍguez et al., 2018; Duan and Paulson, 2020). A recent manuscript demonstrated that sialoglycans on pancreatic cancer cells can engage Siglec-7 and Siglec-9 receptors on tumor-associated macrophages and thereby promote cancer progression (Rodriguez et al., 2021). Interactions of Siglec-10 on TAMs with sialoglycans have shown to promote cancer (Barkal et al., 2019). In addition, blockade of GD-2, a new Siglec-7 ligand, enhanced antitumor immunity in combination with CD47 blockade (Theruvath et al., 2022). Here, we further demonstrate that Siglecs might play a role in antigen processing and presentation and targeting sialoglycan–Siglec interactions in cancer could potentially influence the anti-tumor immunity mediated by DCs.

The limitation of our analysis is mainly the *in vitro* character of our functional studies. Further analysis is certainly needed in animal models that can support a functional role for Siglec receptors on DCs. Moreover, the exact molecular mechanisms on how improved antigen processing and T cell stimulation are mediated in Siglec-deficient DCs remain also elusive and require more work.

In summary, we found several inhibitory Siglec receptors expressed on cDCs in primary cancer samples. Inhibitory Siglecs on cDCs can modulate DC activation, antigen processing, and T cell activation. Our findings provide new insights into mechanisms involved in Siglec-mediated immune escape in cancer.

## Data Availability

The datasets presented in this study can be found in online repositories. The names of the repository/repositories and accession number(s) can be found as follows: https://www.ncbi.nlm.nih.gov/geo/, GSE190700.
