# Supplementary material for: Siglec Receptors Modulate Dendritic Cell Activation and Antigen Presentation to T Cells in Cancer
Source: Front Cell Dev Biol. 2022 Mar 3;10:828916. doi: 10.3389/fcell.2022.828916 (PMC8927547; doi:10.3389/fcell.2022.828916)
Supplement: Supplementary file 1 [file DataSheet1.pdf]

Supplementary data

Siglec receptors modulate dendritic cell activation and antigen presentation to T cells

Jinyu Wang, Michela Manni, Anne Bärenwaldt, Ronja Wieboldt, Nicole Kirchhammer, Michal Stanczak, Alfred Zippelius, David König, Natalia Rodrigues Manutano, and Heinz Läubli

Material

REAGENTS or RESOURCE	SOURCE	IDENTIFIER
**Antibodies**		
APC-eFluor780 anti-human CD3 (SK7)	eBioscience	Cat# 47-0036-42
APC-eFluor780 anti-human CD19 (SJ25C1)	eBioscience	Cat# 47-0198-42
APC-eFluor780 anti-human CD56 (CMSSB)	eBioscience	Cat# 47-0567-42
BV711 anti-human CD45 (H130)	Biolegend	Cat# 304050
PE-cy7 anti-human CD11c (3.9)	Biolegend	Cat# 301608
FITC anti-human HLA-DR (L243)	Biolegend	Cat# 307604
PE-CF594 anti-human CD123 (7G3)	BD	Cat# 562391
BV421 anti-human CD1c (L161)	Biolegend	Cat# 331526
Percp-cy5.5 anti-human CD141 (M80)	Biolegend	Cat# 344112
PE anti-human Siglec-7 (6-434)	Biolegend	Cat# 339204
APC anti-human Siglec-8 (7C9)	Biolegend	Cat# 347106
APC anti-human Siglec-9 (191240)	R&D Systems	Cat# FAB1139A-100
PE anti-human Siglec-10 (5G6)	Biolegend	Cat# 347604
BV421 anti-mouse F4/80 (BM8)	Biolegend	Cat# 123132
BV421 anti-mouse GR-1 (RB6-8C5)	Biolegend	Cat# 108433
Percp-cy5.5 anti-mouse CD45 (30-F11)	eBioscience	Cat# 45-0451-82

BV711 anti-mouse CD11c (N418)	Biolegend	Cat# 117349
BV605 anti-mouse MHC-II (M5/114.15.2)	Biolegend	Cat# 107639
PE-cy7 anti-mouse MHC-II (M5/114.15.2)	eBioscience	Cat# 25-5321-80
APC anti-mouse MHC-II (M5/114.15.2)	Biolegend	Cat# 107614
BV510 anti-mouse CD11b (M1/70)	Biolegend	Cat# 101263
AF488 anti-mouse CD8a (53-6.7)	BD Biosciences	Cat# 557668
APC anti-mouse CD8 (53-6.7)	eBioscience	Cat# 17-0081-83
AF488 anti-mouse CD103 (2E7)	Biolegend	Cat# 121420
PE-cy7 anti-mouse Siglec-E (M1304A01)	Biolegend	Cat# 677108
PE anti-mouse Siglec-F (E50-2440)	BD Biosciences	Cat# 552126
APC anti-mouse Siglec-G (SH2.1)	eBioscience	Cat# 17-5833-82
PE anti-mouse MHC-I (AF6-88.5)	Biolegend	Cat# 116508
APC anti-mouse CD40 (3/23)	BD Biosciences	Cat# 558695
BV605 anti-mouse CD80 (16-10A1)	Biolegend	Cat# 104729
PE anti-mouse CD80 (16-10A1)	Biolegend	Cat# 104708
PE anti-mouse CD83 (Michel-19)	Biolegend	Cat# 121507
APC anti-mouse CD86 (GL-1)	Biolegend	Cat# 105012
PE anti-mouse CD86 (GL-1)	Biolegend	Cat# 105008
PE-cy7 anti-mouse CD3 (145-2C11)	BD Biosciences	Cat# 552774

BV605 anti-mouse CD3 (145-2C11)	Biolegend	Cat# 100351
APC anti-mouse CD4 (GK1.5)	eBioscience	Cat# 17-0041-83
BV711 anti-mouse CD4 (GK1.5)	BD Horizon	Cat# 563050
FITC anti-mouse CD25 (PC61)	Biolegend	Cat# 102006
Continued		

REAGENTS or RESOURCE	SOURCE	IDENTIFIER
**Antibodies**		
Percp-cy5.5 anti-mouse CD44 (IM7)	Biolegend	Cat# 103032
PE anti-mouse CD69 (H1.2F3)	Biolegend	Cat# 104522
Percp-cy5.5 anti-mouse CD69 (H1.2F3)	eBioscience	Cat# 12-0691-83
**Chemicals Dyes**		
CellTrace CFSE Cell Proliferation Kit	Invitrogen	Cat# C34554
CellTrace Violet Cell Proliferation Kit	Invitrogen	Cat# C34557
Zombie UV Fixable Viability Kit	Biolegend	Cat# 423108
Zombie NIR Fixable Viability Kit	Biolegend	Cat# 423106
Fixable Viability Dye eFluor 450	eBioscience	Cat# 65-0863-14
**Proteins**		
Recombinant murine M-CSF	Peprotech	Cat# 315-02
Recombinant murine GM-CSF	Peprotech	Cat# 315-03
EndoFit Ovalbumin	Invivogen	Cat# Vac-pova
**Critical commercial assays**		
EasySep Mouse CD4+ T Cell Isolation Kit	Stemcell Technologies	Cat# 19852
EasySep Mouse CD8+ T Cell Isolation Kit	Stemcell Technologies	Cat# 19853

EasySep Mouse CD11c Positive Selection Kit II	Stemcell Technologies	Cat# 18780
Mouse Cytokine Array / Chemokine Array 44-plex (MD44)	Eve Technologies	
RNeasy Plus Micro Kit	Qiagen	Cat# 74034

Supplementary Figures

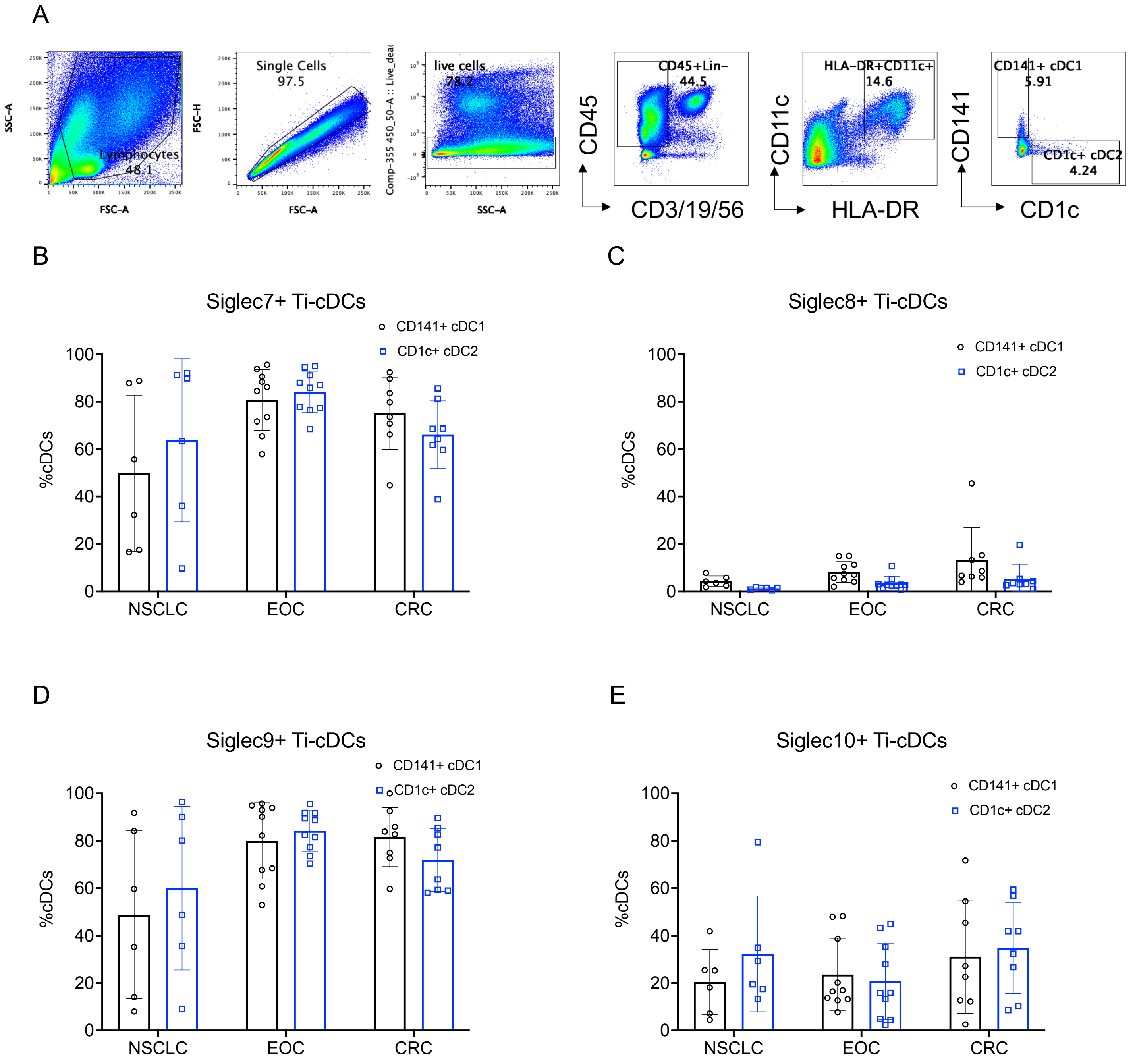

Figure S1 Human intratumoral conventional dendritic cell subsets gating and expression of inhibitory Siglecs. A, Gating strategy. B-E, Expression patterns of inhibitory Siglecs on tumor-infiltrating cDCs (Ti-cDCs) from NSCLC, EOC and CRC patient samples. (B) Siglec-7, (C) Siglec-8, (D) Siglec-9 and (E) Siglec-10.

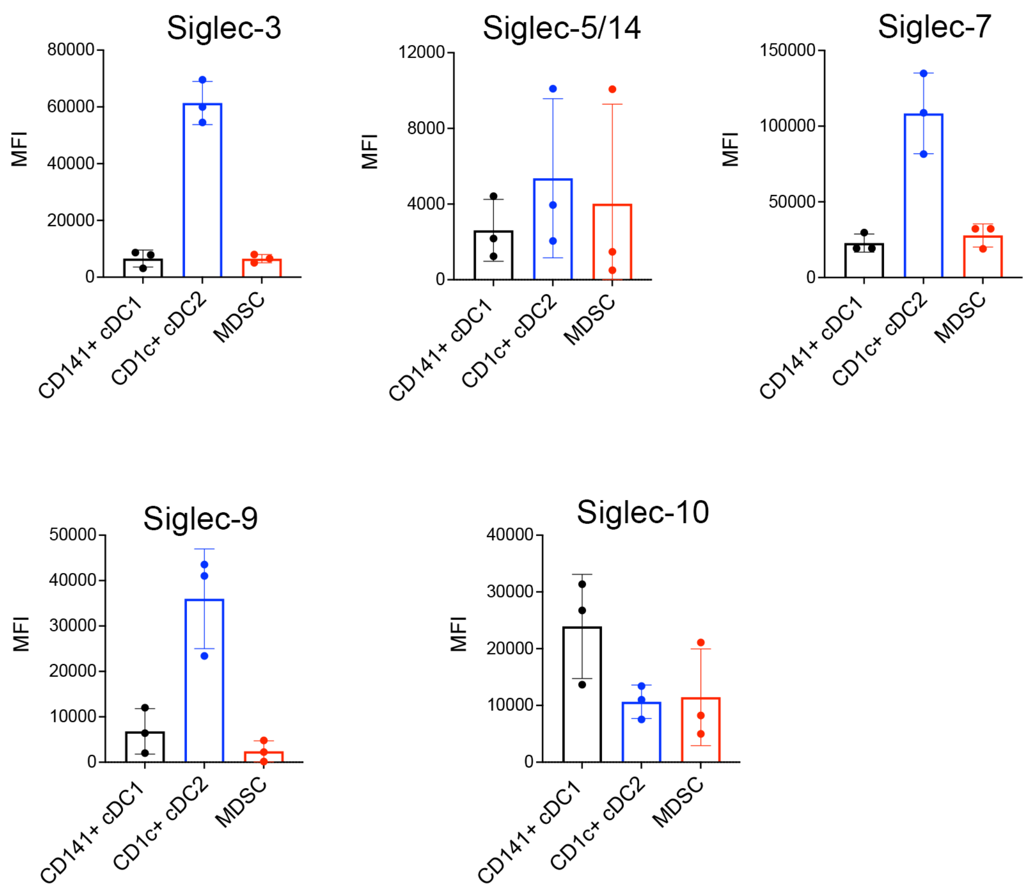

Figure S2 Expression of inhibitory Siglecs on intratumoral DCs and other myeloid cells. Intratumoral cells were stained and gated as shown in Figure S1. Different dendritic cells were analyzed for their expression of the main inhibitory receptors.

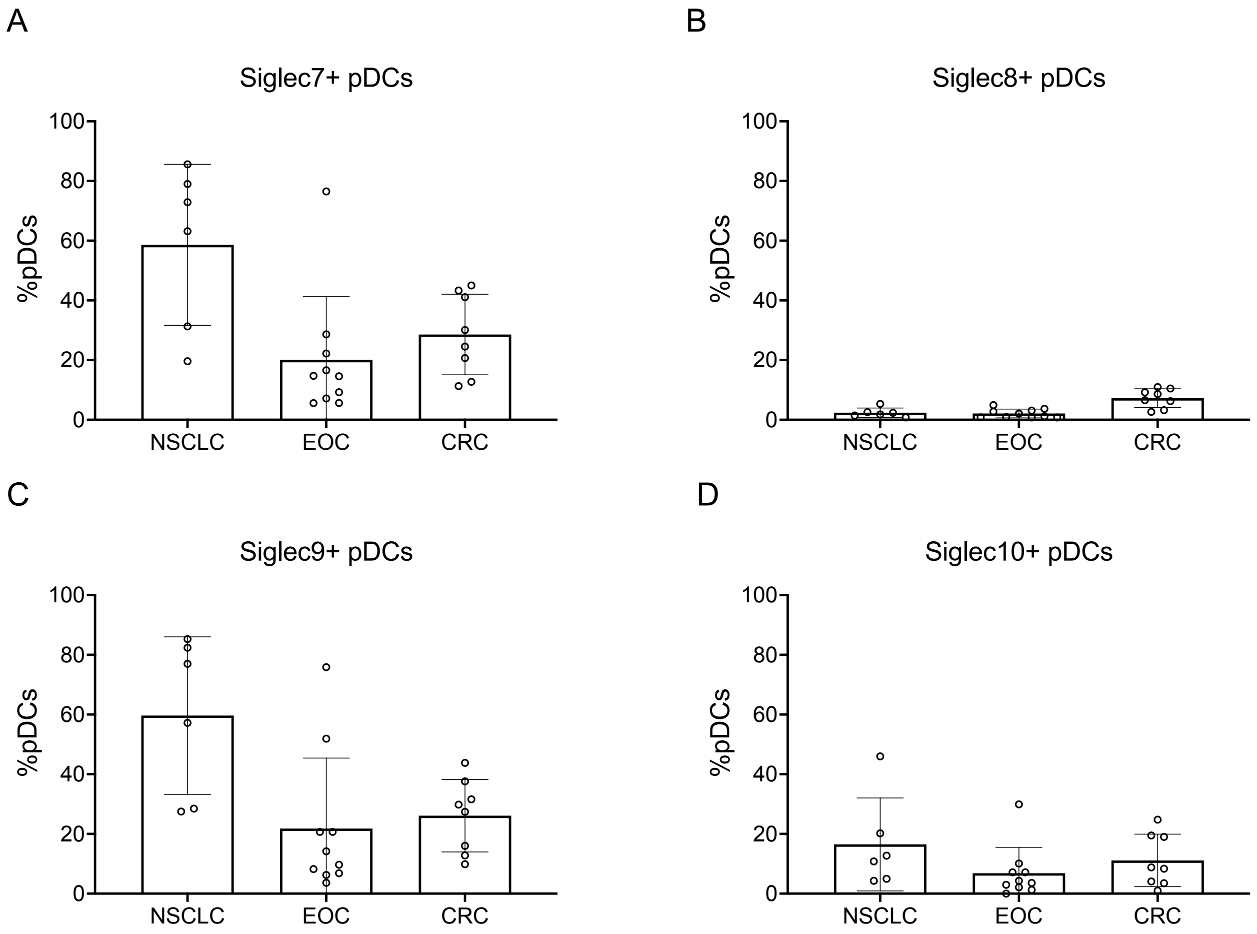

Fig S3 Expression of inhibitory Siglecs on human tumor-infiltrating pDCs. A-D, Flow cytometry analysis of the expression of inhibitory Siglecs on plasmacytoid dendritic cells (pDC) from primary tumor samples of non-small cell lung cancer (NSCLC), epithelial ovarian cancer (EOC) and colorectal cancer (CRC) patients. Data are presented as mean (± SD).

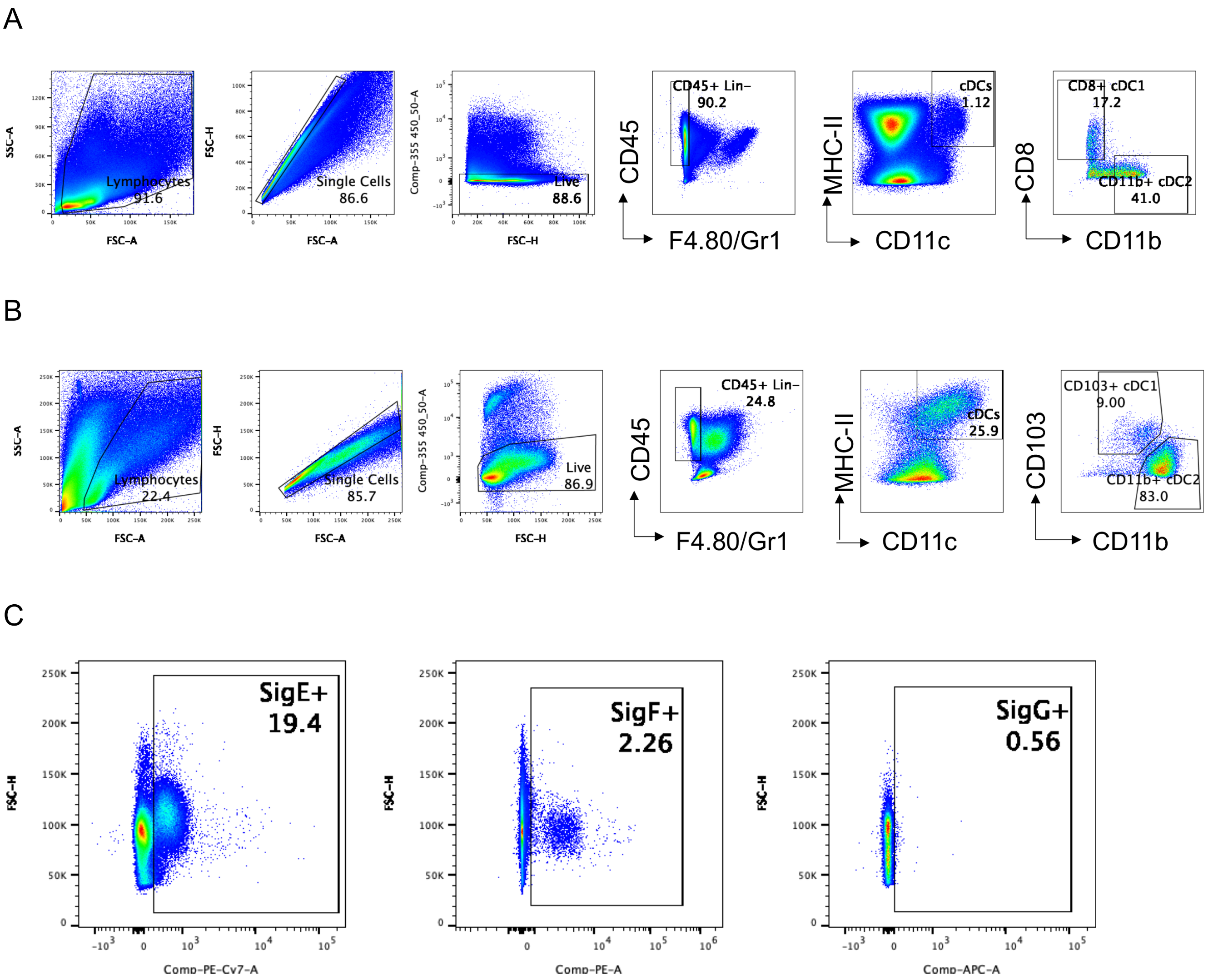

Figure S4 Gating strategy of mouse cDCs and Siglecs. A, and B, Mouse cDC subsets gating strategies of samples from (A) spleens and (B) tumors. (C) Gating for each inhibitory murine Siglec.

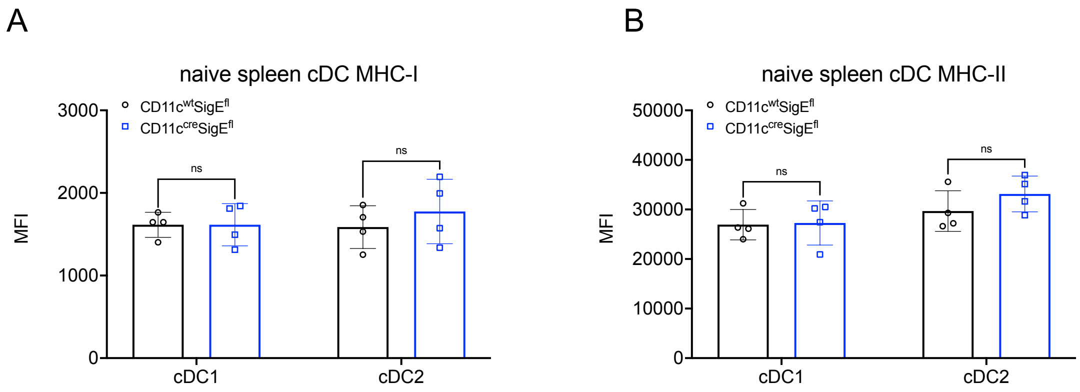

Figure S5 Maturation markers of naïve spleen cDCs from ELox mice and littemates. A, and B, Expression levels of (A) MHC-I and (B) MHC-II molecules on naïve spleen cDCs.

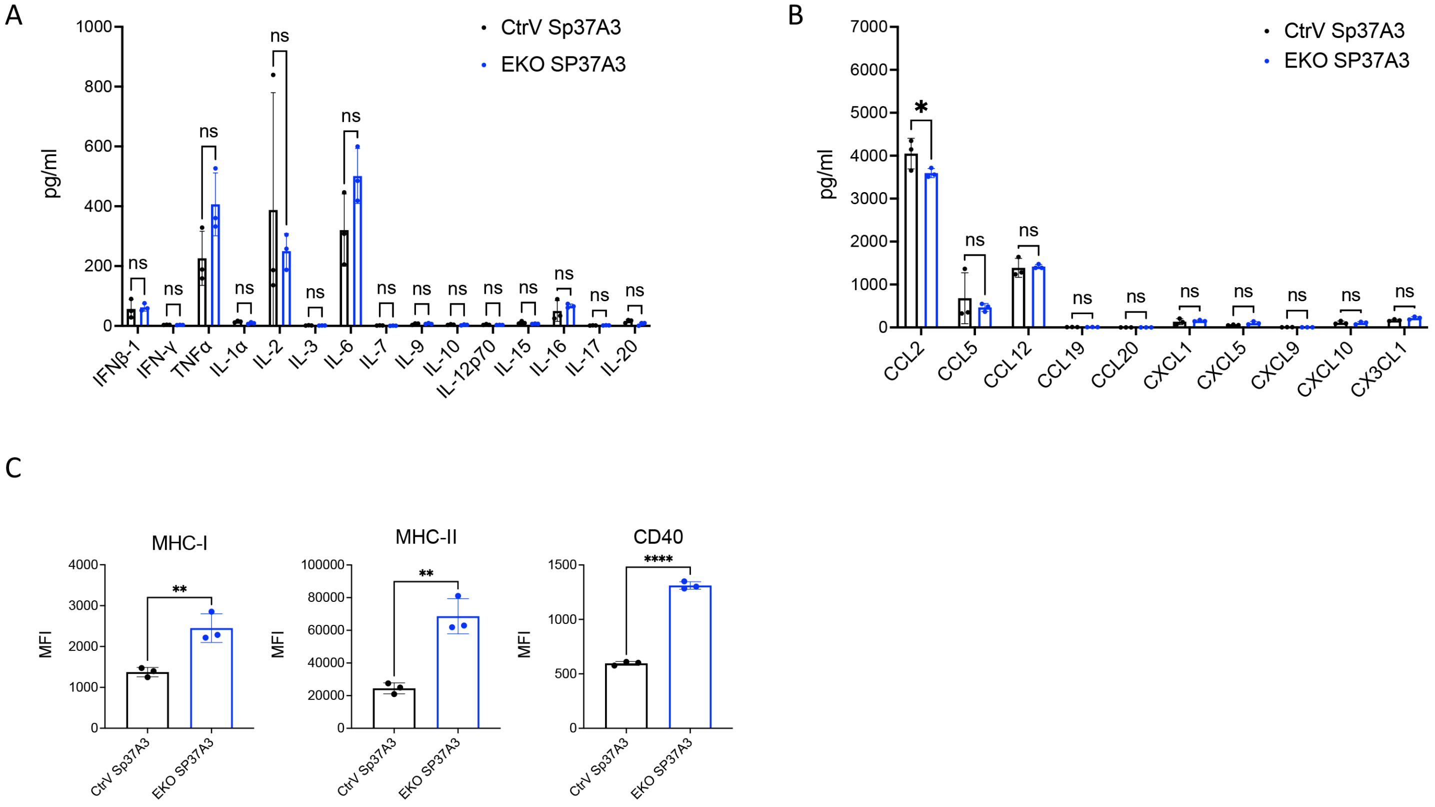

Figure S6 Siglec-E-deficient DCs demonstrate elevated maturation status upon stimulation. A-C, Secretion levels of other cytokines (A), chemokines (B) and maturation markers (C) of CtrV and EKO Sp37A3 cells. Data are presented as mean (± SD), and unpaired t test was used for one-way comparisons (*P<0.0332, **P<0.0021, ***P<0.0002, ****P<0.0001).

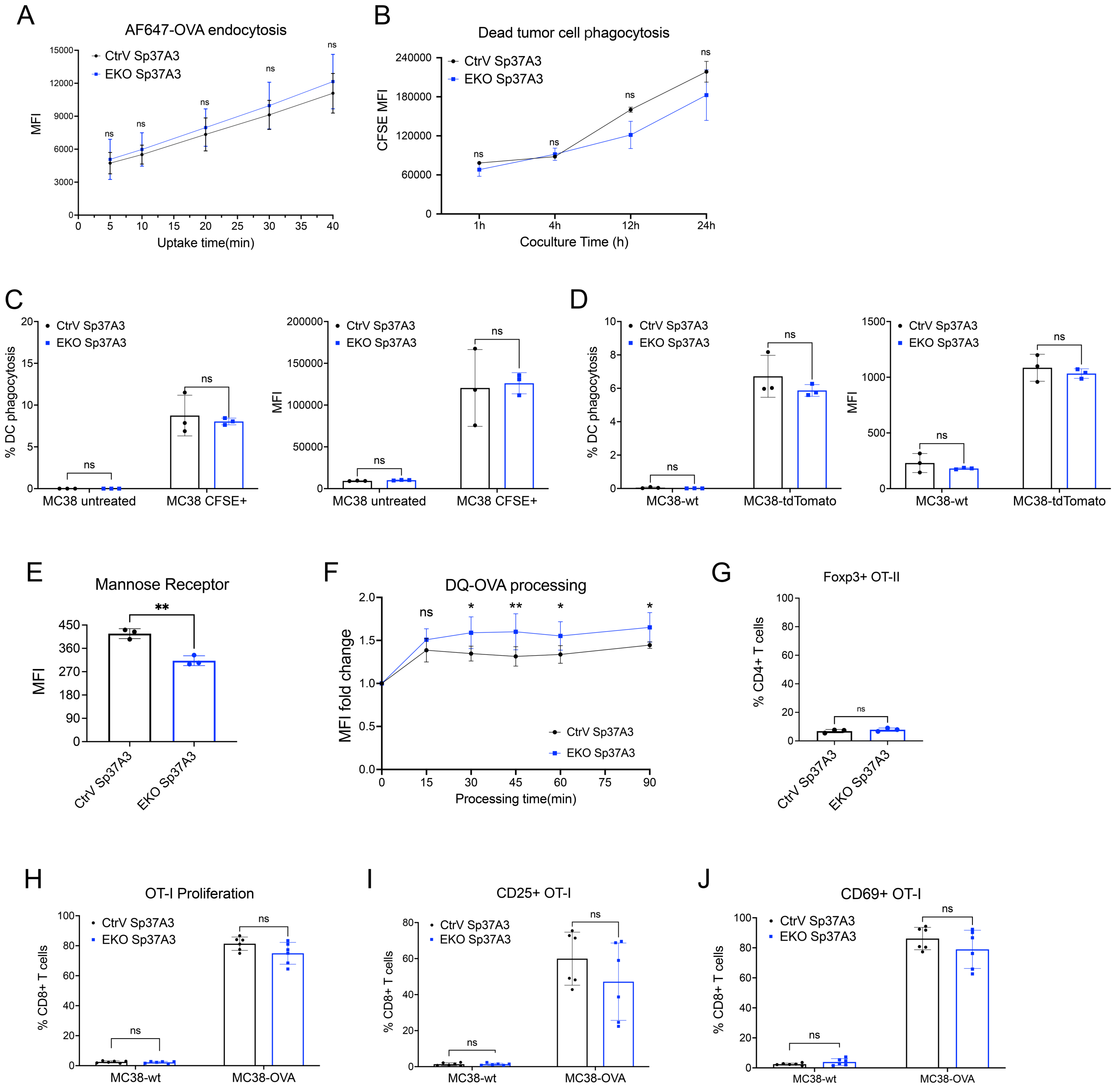

Figure S7 Siglec-E expression affects antigen processing. A, and **B**, Phagocytosis of soluble AF647-conjugated OVA antigen (A) and CFSE-labelled heat-shocked MC38 cells (B) by Sp37A3 DCs. **C**, and **D**, Sp37A3 endocytosis of live fluorescent tumor cells. Sp37A3 DCs were co-cultured 4 hours at 1:2 ratio with untreated MC38 cells or CFSE-labelled MC38 cells (C), wildtype MC38 (MC38-wt) cells or tdTomato+ MC38 cells (D). **E**, Expression level of Mannose Receptor on both Sp37A3 cells. **F**, DQ-OVA antigen processing assay. EKO Sp37A3 cells were compared with CtrV Sp37A3 cells. **G**, Frequency of Foxp3+ Treg cells in CD4+ OT-II cells. **H-J**, Sp37A3 DCs were pulsed with heat-shocked wildtype

MC38 cells or MC38-OVA cells. Then co-culture with OT-I cells for 48h to test antigen cross-presentation efficiency. Proliferation was measured by flow cytometric determination of cell trace violet dilution (H). In addition, activation of T cells was measured by CD25 (I) and CD69 (J) expression studied by flow cytometry. Data are presented as mean (± SD) and two-way ANOVA was used for two-way comparisons (*P<0.0332, **P<0.0021, ***P<0.0002, ****P<0.0001).

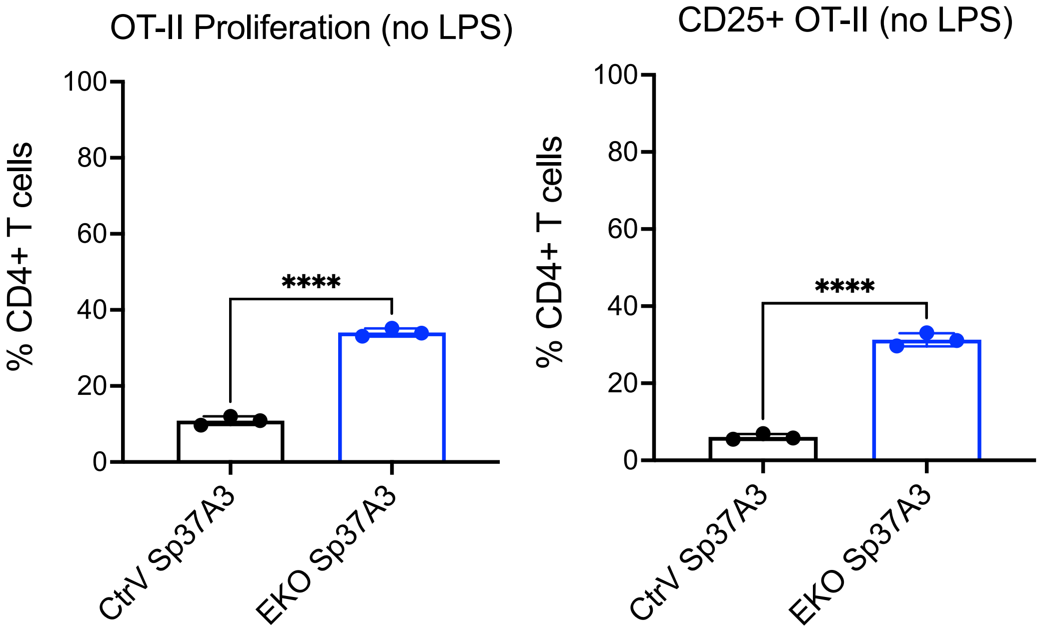

Figure S8 Proliferation and activation of OT-II cells without LPS used for DC activation. Experiments were performed similarly as studies shown in main figure 5, but no LPS was used for maturation of DC cell lines.
